# Culturally relevant settings for TB transmission in an African city with endemic TB

**DOI:** 10.5588/ijtldopen.25.0275

**Published:** 2025-12-10

**Authors:** T.H.T. Quach, R. Kakaire, S. Zalwango, J.N. Sekandi, M.E. Castellanos, C.C. Whalen, N. Kiwanuka

**Affiliations:** 1 Global Health Institute, College of Public Health, University of Georgia, Athens, GA, USA;; 2 Department of Public Health and Environment, Kampala Capital City Authority, Kampala, Uganda;; 3 Public Health and Tropical Medicine, College of Public Health, Medical and Veterinary Sciences, James Cook University, Townsville, QLD, Australia;; 4 Department of Epidemiology and Biostatistics, School of Public Health, College of Health Sciences, Makerere University, Kampala, Uganda.

**Keywords:** tuberculosis, community transmission, latent tuberculosis infection, TBI

## Abstract

**BACKGROUND:**

TB persists because *Mycobacterium tuberculosis* (*M. tb*) is transmitted through unknown community contact networks. This transmission occurs in culturally relevant settings as infectious cases go about their daily lives. We performed a prospective cohort study in Kampala, Uganda, to estimate incident TB infection (TBI) and relate it to time spent in a broad array of settings.

**METHODS:**

In a prospective cohort study of 1,275 adult residents without TBI, we measured cumulative incidence of infection at 1 year using the tuberculin skin test. We measured time spent in community settings using a validated questionnaire and related settings to incident infection using Poisson regression analysis.

**RESULTS:**

The settings visited by participants varied by weekday and sex. Participants spent most of their time at home or work regardless of the weekday. New infection with *M. tb* was associated with worship centres, schools, and homes of relatives or friends, though these effects were modified by day of the week, categorised as weekday or weekend.

**CONCLUSION:**

Social settings, such as schools or worship centres, may be appropriate sites to screen for TB. Community-based interventions to control TB should consider age, sex, and day of the week to reduce gaps in coverage.

TB is present in every country around the world, but the greatest burden is found in lower-income countries in the southern hemisphere. The disease persists because one case is replaced by at least one other case over time.^1^ Indeed, by the time prevalent cases are detected and properly treated, the next generation of cases have already been infected. At a population level, the epidemic of TB can only be controlled by reducing transmission or mitigating progression following infection. *Mycobacterium tuberculosis* (*M. tb*) is transmitted to close contacts of infectious TB cases in the households or social networks of index cases. In endemic settings, most transmission appears to occur in the community in undefined contact networks. In Kampala, Uganda, an African city with endemic disease, most of the infection with *M. tb* has been attributed to exposures in the community, outside of households or reported social networks of index cases.^2^ When tracking incident cases of TB using molecular epidemiology, a similar pattern emerges. Most demonstrated transmission occurs between cases that cannot be linked via contact investigation or epidemiologic analysis.^3^ Transmission of *M. tb* requires adequate contact between an infectious case and one or more susceptible contacts.^4,5^ Although adequate contact may take many forms, one necessary feature is co-location of the case and contact. In a household or social network, the locations of mixing may be readily ascertained, whereas in the community, the locations of mixing between cases and casual or incidental contacts may be difficult to identify. Since most transmission of *M. tb* occurs in the community, a better understanding of how cases and contacts mix is needed to develop community-based interventions tailored to settings where co-location occurs.

We propose a theoretical framework wherein *M. tb* transmission occurs in culturally relevant settings that are characterised by their intended use and their built environment. As index cases go about their daily lives, they transit through various settings and create a trajectory of locations where they mix with susceptible contacts. This network of settings and locations may be a surrogate measure of transmission in the community. If these settings could be identified and mapped, it would be possible to design interventions around them. In this project, we performed a prospective cohort study in Kampala, Uganda, to estimate incident TB infection (TBI) and relate it to time spent in culturally relevant settings.

## METHODS

We conducted a prospective cohort study among residents of Lubaga division of Kampala, Uganda, from June 2014 to October 2017.^6^ Adults aged from 17 to 60 years old who had a tuberculin skin test (TST) less than 5 mm at the time of screening and no signs or symptoms of TB were enrolled and evaluated at 1 year for incident infection defined as TST conversion. Because of delays in recruitment, the duration of follow-up was extended up to 2 years among participants who had not converted the TST by 1 year. The sample size was determined to estimate the annual incidence of TBI of 5%, 80% power, and 5% error. Statistical significance was set at 0.05 level.

### Data collection

At quarterly intervals starting at baseline, we measured time spent in various settings as our exposure. We characterised time in settings as reported in a typical week, partitioned as weekday and weekend. This information was collected using a validated, pictorial survey.^7^ The survey included pictures of common settings in the city, including home, homes of others (e.g., friends or relatives), workplace, school, worship centre, social gathering places, bar, shopping sites, hair salon, gym and/or non-athletic recreation, hospital or clinic, and public transportation. Demographic and epidemiologic characteristics were obtained through structured interviews at baseline. HIV testing was performed following the Uganda Ministry of Health National Testing algorithms. TST was measured using the Mantoux method,^6^ and standard definitions for a positive test were used.^8^ Participants who did not meet the definition of a positive TST were classified as non-converters.

### Statistical analysis

Cohort characteristics were summarised with proportions and measures of central tendency. We calculated time spent at each setting as frequency and duration. For frequency per setting, we aggregated reports for each setting across the entire cohort for all evaluations and expressed them as a percentage of the total number of evaluations (N = 6,439). For duration, we estimated the proportion of the day spent at each setting by aggregating the duration of time spent at each setting across the entire cohort for all evaluations and by expressing time spent at each setting as a percentage of total hours. These analyses were stratified by sex, age groups, and TST conversion and examined differences by two-proportion z-test. Further, we estimated the median and interquartile range (IQR) of duration of time spent at a setting when it was reported by the participant across baseline and quarterly evaluations and examined differences between weekday/weekend by the Mann–Whitney *U* test. A Poisson regression model with robust standard errors (R version 3.6.1) and an offset for observation time was used to estimate the rate ratio (95% confidence intervals [CIs]) for the association between TST conversion and time spent at a setting. To fit the model, we used the mean duration of time spent per participant across the evaluations and multivariable models for each setting separately, adjusting for age, sex, religion, and exposure to a TB case within 1 year.^6^ As a sensitivity analysis, we used the maximum duration of time spent per participant in each setting to fit the Poisson regression model.

### Ethical statement

The study was approved by the University of Georgia Institutional Review Board, the Higher Degrees Research and Ethics Committee at Makerere University School of Public Health, and the Uganda National Council for Science and Technology.

## RESULTS

Of 1,681 enrolled participants, 1,275 (76%) were available with complete TST outcome information. The cohort included adults with a median age of 24 years (IQR: 20, 29), of whom most were women (59%; Table 1). HIV infection was present in 5% of participants. Most participants had received Bacille Calmette-Guérin (BCG) vaccination. Some participants (9%) were recently exposed to TB patients. The 1,275 participants reported a total of 6,439 evaluations to different settings, including 1,274 at baseline, 1,242 at 3-month follow-up, 1,202 at 6-month follow-up, 1,208 at 9-month follow-up, 1,142 at 12-month follow-up, and 371 beyond 12 months (Supplementary Data Figure S1). Of the 1,681 eligible participants, 406 (24%) did not have a repeated TST because of refusal or lost-to-follow-up.

**Table 1.** Characteristics of 1,275 participants with available tuberculin skin test (TST) at follow-up.

Characteristics	Frequency (%)	Person-year of observation
Age in years, median (IQR)	24 (20, 29)	1,468
Age category		
17–21	398 (31.2)	461 (31.4)
22–29	585 (45.9)	664 (45.2)
30–60	292 (22.9)	343 (23.4)
Sex		
Female	747 (58.6)	854 (58.2)
Male	528 (41.4)	614 (41.8)
Marital status		
Married	707 (55.5)	810 (55.1)
Never married	568 (44.5)	659 (44.9)
Education level (n = 1,272)		
None or primary	406 (31.9)	480 (32.7)
Secondary/post-secondary	866 (68.1)	986 (67.3)
Monthly income in USD (n = 1,271)		
27 or less than	453 (35.6)	520 (35.5)
>27 to <54	383 (30.1)	436 (29.8)
54 or more	435 (34.2)	508 (34.7)
Religion (n = 1,273)		
Roman Catholic	518 (40.7)	602 (41.1)
Others	755 (59.3)	864 (58.9)
Smoking (n = 1,268)		
Never smoked	1,219 (96.1)	1,404 (96.2)
Former/current smoker	49 (3.9)	56 (3.8)
Alcohol use (n = 1,256)		
Non-users	989 (78.7)	1,143 (79.0)
Light users	192 (15.3)	215 (14.9)
Heavy users	75 (6.0)	88 (6.1)
BCG vaccination		
No/unknown	99 (7.8)	112 (7.6)
Probably yes	147 (11.5)	171 (11.6)
Yes	1,029 (80.7)	1,186 (80.7)
HIV test at baseline		
Negative	1,205 (94.5)	1,389 (94.6)
Positive	61 (4.8)	70 (4.8)
Refused	9 (0.7)	9 (0.6)
Exposure to known case within 1 year (n = 1,268)		
No	1,158 (91.3)	1,332 (91.2)
Yes	110 (8.7)	128 (8.8)

**Figure 1.**
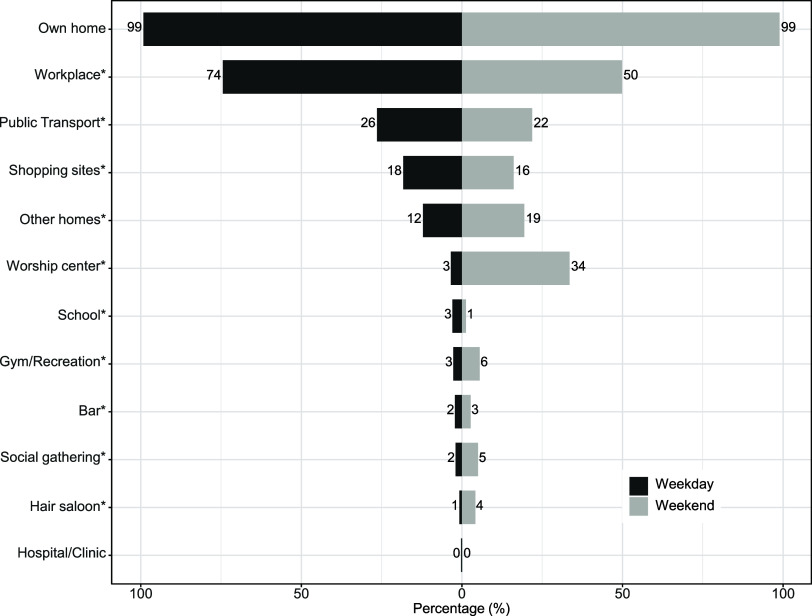
Percent of evaluations when participants reported their attendance at a setting weekday/weekend. *P < 0.05 difference between weekday and weekend.

Of the 1,275 participants, 194 converted over study period of whom 158 converted during the first year (previously published^6^), yielding an annual risk of infection of 12.4%. Participants reported spending most of their time at home regardless of whether it was a weekday or weekend (Figure 1). Participants spent a median of 12 h at home on weekdays and 15 h on weekends (Supplementary Data Figure S3). The workplace was the next most frequent setting reported where 74% of the participants reported spending time at their workplace; on weekends, this was reduced to 50%. The median time at work when reported was 11 h on weekdays (IQR: 10, 12) and 10 h (IQR: 8, 12) on weekends (Supplementary Data Figure S3; *P* < 0.001). When stratifying by age category and sex, we found consistent patterns, where men more often attended their workplace during weekdays and weekends than women (Supplementary Data Figure S2A); on weekends, both men and women spend less time at work.

Apart from home and the workplace, public transport, shopping sites, and other homes were listed as settings visited on weekdays by participants in 12% to 26% of the evaluations (Figure 1) for shorter periods of time (Supplementary Data Figure S3). Women reported recent exposure to TB cases in shopping sites and the homes of relatives of friends. On weekends, participants engaged other settings, including churches or mosques, gyms, bars, medical facilities, other homes, and social gatherings. Although these settings were reported more often on weekends, the duration spent at each location was variable but generally less than 5 h. Among these settings, men reported known exposures to TB cases at bars more often than women.

**Figure 2.**
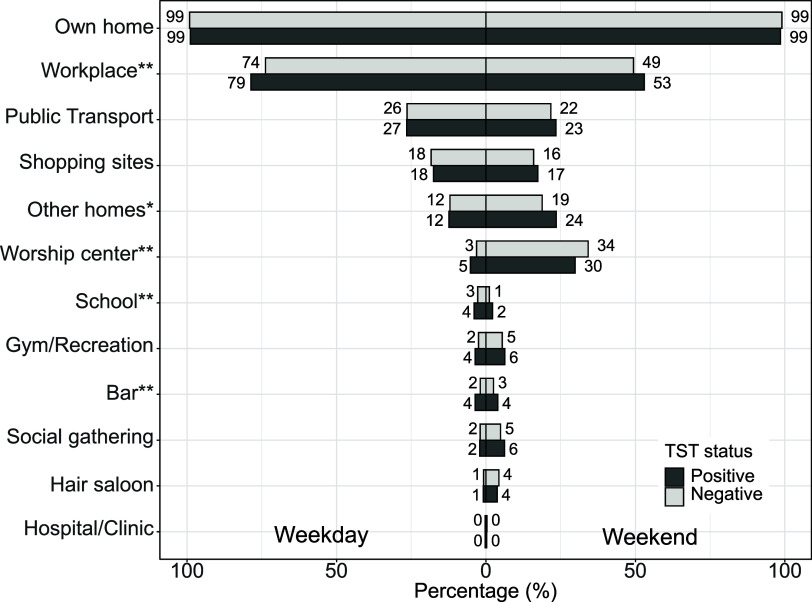
Percent of evaluations when participants reported their attendance at a setting stratified by TST status and weekday/weekend. **P < 0.05 between TST converters and non-converters on both weekday and weekend. *P < 0.05 between TST converters and non-converters on weekend only.

**Figure 3.**
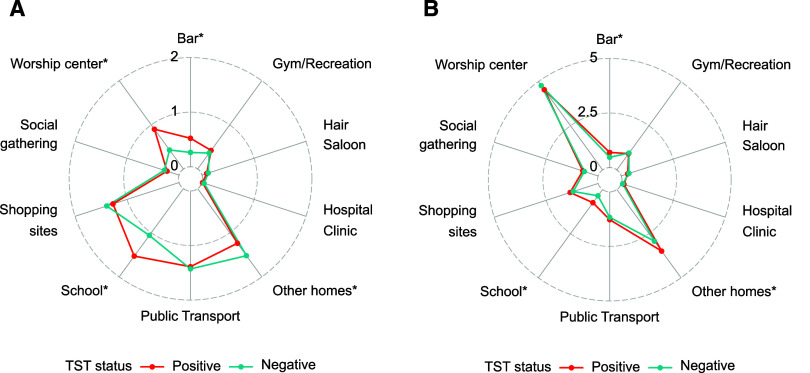
Radial graph showing the percent of the day spent at various settings on **A:** weekdays and **B:** weekends, stratified by TST status. *P < 0.05 between TST converters and non-converters.

The risk of TST conversion during follow-up was greater among participants who spent a greater proportion of their time at their workplace for both weekdays and weekends (Figure 2 and Supplementary Data Figure S7), though there was variation in duration of time spent in the workplace (Supplementary Data Figure S4); a similar pattern of risk was found for spending time at school or in bars (Figures 2 and 3), though these venues comprised only a short period of time per week. Participants experienced greater risk of infection when they spent more time visiting the homes of relatives or friends during the weekends or when they spent more time at their place of worship during the weekdays (Figure 3).

In a multivariable Poisson regression model, worship centres, schools, and homes of relatives or friends were associated with new TBI (Table 2), though these effects were modified by day of the week, categorised as weekday or weekend. In the case of schools, the risk was greater among participants who spent time in school during the weekend as compared with those who attended school only during the weekday (rate ratio [RR] = 1.19 vs. 1.1; Table 2). As for worship centres, the risk was present only among worshippers who spent time on weekdays in the place of worship (RR = 1.14; Table 2). In homes of relatives or friends, the risk was greater on the weekend (RR = 1.12; Table 2). The patterns of risk varied by weekday or weekend for other settings, but the interaction was not statistically significant. Using maximum duration of time spent as a summary measure, staying at own home was associated with a lower risk of acquiring TBI (RR = 0.95; 95% CI: 0.92, 0.98).

**Table 2.** Multivariable Poisson regression models to evaluate effect modification by weekday or weekend, adjusting for age, sex, and religion.

Settings	Mean duration of weekday visits	Mean duration of weekend visits
	aRR (95% CI)	
Own home	0.99 (0.95, 1.04)	0.99 (0.94, 1.03)
Other homes	0.94 (0.77, 1.14)	1.13 (1.01, 1.26)
Workplace	0.99 (0.95, 1.04)	0.99 (0.95, 1.03)
School	1.11 (1.02, 1.20)	1.20 (1.05, 1.36)
Worship centre	1.14 (1.07, 1.23)	0.99 (0.87, 1.13)
Social gathering	0.75 (0.45, 1.28)	1.07 (0.87, 1.31)
Bar	1.02 (0.79, 1.32)	0.95 (0.74, 1.21)
Shopping sites	0.88 (0.68, 1.14)	1.08 (0.84, 1.38)
Hair salon	0.77 (0.31, 1.88)	0.91 (0.41, 2.03)
Gym/recreation	1.04 (0.72, 1.50)	0.94 (0.73, 1.22)
Hospital/clinic	0.29 (0.01, 8.39)	1.12 (0.72, 1.73)
Public transport	0.91 (0.62, 1.32)	1.17 (0.82, 1.67)

aRR = adjusted relative risk

## DISCUSSION

The aim of this study was to relate incident infection with *M. tb* to the settings where adults spend their time in an African city with endemic TB. The central finding was that new *M. tb* infection was likely spread in both public and private settings outside of the home. In community settings, such as workplaces, worship centres, schools, and bars, the likelihood of infection varied during the week according to the settings visited. In some settings, such as workplace and bars, the risk was present throughout the week, whereas for worship centres, the risk was elevated either during weekdays or on the weekend. In private settings, the likelihood of infection was greater in the homes of relatives or friends only during the weekend. When accounting for demographics, religious practice, and reported exposure to a TB case, the risk of infection was associated with schools,^9^ places of worship, and homes of relatives and friends. Moreover, as suggested by the descriptive analysis, there was effect modification by weekday or weekend with each of these settings.^10^

In the community settings, the risk of infection was elevated in schools, regardless of the day of the week, but was higher among students who spent time at school on weekends. For worship centres, the risk was present only among the parishioners who visited their place of worship during weekdays. In private settings, the risk of incident infection was lower in participants who spent more time at home, an effect that was best seen in the Poisson regression analysis of the maximal time per day at home. Since most homes in Kampala do not contain an infectious case of TB, the risk of acquiring infection is low in those homes. Socialising in the homes of family or friends on weekends was, however, associated with a higher risk of infection. These findings are consistent with other studies which indicate that transmission of TB occurs more often outside of the home in the community.^6,11–14^

Taken together, there are strong social and cultural factors that influence where people spend their time in urban sub-Saharan Africa. The settings themselves may be where exposures to undetected infectious cases occurred; alternatively, these settings may be markers of behaviours that lead to exposures in the community. In either case, our findings may have important implications for the design and conduct of community interventions for TB control. Schools and religious gatherings, for example, may open gateways for engaging the community in TB control since they have an established infrastructure with trusted leadership. Although there may be many schools and religious centres in Kampala city, they are countable, and their locations are known unlike bars or homes of relatives. These sites may be amenable to screening for disease and infection using mobile health vans. Moreover, since the inscape of these settings is to provide instruction or education, they may be ideal venues for public health education about TB. Any interventions should coordinate closely with the national TB programme and be sensitive to the potential stigma of TB in the community.

Our findings also suggest that community interventions may need to be designed differently for men and women to avoid gaps in coverage. For example, one approach to active case finding in the community is to perform house-to-house evaluations in areas with high disease prevalence or ‘hotspots’.^15^ This approach may, however, preferentially screen women over men because women spend more time at home. On balance, no single type of intervention based on settings will suffice to cater for all at-risk populations in a fully equitable manner. TB control programmes should integrate multiple interventions to access different populations at risk through the settings they frequent. These interventions could be performed during work hours or in the evenings, on weekdays or weekends, and may be repeated at intervals. They may involve a combination of household contact tracing and screening in schools or religious settings to identify unidentified cases in the community. To identify the most relevant settings for evaluation in each community, public health workers could engage stakeholders and members of the community. Through community-based participatory research,^16^ health officials may draw upon their knowledge of the local TB epidemiology and integrate it with the intimate knowledge of the community as contributed by its members and stakeholders. This approach would encourage the buy-in from the community, dispel myths about TB, create more direct pathways to diagnosis and treatment, educate health providers, and build a foundation for sustainability.

Despite the size of the cohort and duration of observation, we were unable to estimate the risk of infection in some settings that were infrequently reported, such as medical settings. We did not find workplace to be associated with infection either, though it was a common setting overall. Since we aggregated workplace settings, it is possible that we did not identify some types of workplaces where TB transmission occurs. Another limitation is that we did not ascertain the geographic locations of each setting. While a setting may describe a built environment with shared airspace, it does not usually provide the location. For the purposes of community interventions, geographic location may be more important than setting in tailoring the intervention.^17^

Residents of African cities visit many types of settings as they go about their daily lives. The settings which varied by day of the week and sex may have implications for the transmission of *M. tb*. In general, community-based interventions to control TB should account for different settings to minimise the gaps in coverage. This approach may best be achieved through community-based participatory planning and implementation.

## Supplementary Material


