# Supplementary material for: Culturally relevant settings for TB transmission in an African city with endemic TB
Source: IJTLD Open. 2025 Dec 10;2(12):745–50. doi: 10.5588/ijtldopen.25.0275 (PMC12699961; doi:10.5588/ijtldopen.25.0275)
Supplement: Supplementary file 1 [file ijtldopen25-0275_supplementarydata1.pdf]

SUPPLEMENT

Table S1. Baseline characteristics of study population according to complete outcome information

Characteristics	Total	TST Outcome Evaluated		p-value
		Yes (n=1275)	No (n=406)	
Age in years, median (IQR)	24 (20, 29)	24 (20, 29)	24 (20, 29)	0.376
Sex				
Female	1006 (59.8)	747 (58.6)	259 (63.8)	0.056
Male	673 (40.0)	528 (41.4)	145 (35.7)	
(Missing)	2 (0.1)	0 (0.0)	2 (0.5)	
Monthly income in USD				
27 or less than	598 (35.6)	453 (35.5)	145 (35.7)	0.992
>27 to <54	504 (30.0)	383 (30.0)	121 (29.8)	
54 or more	572 (34.0)	435 (34.1)	137 (33.7)	
(Missing)	7 (0.4)	4 (0.3)	3 (0.7)	
Religion				
Roman Catholic	675 (40.2)	518 (40.6)	157 (38.7)	0.552
Others	1002 (59.6)	755 (59.2)	247 (60.8)	
(Missing)	4 (0.2)	2 (0.2)	2 (0.5)	
Marital status				
Married	920 (54.7)	707 (55.5)	213 (52.5)	0.367
Never married	759 (45.2)	568 (44.5)	191 (47.0)	
(Missing)	2 (0.1)	0 (0.0)	2 (0.5)	
Education level				
None or Primary	526 (31.3)	406 (31.8)	120 (29.6)	0.474
Secondary/Post-secondary	1148 (68.3)	866 (67.9)	282 (69.5)	
(Missing)	7 (0.4)	3 (0.2)	4 (1.0)	
BCG vaccination				
No/Unknown	134 (8.0)	99 (7.8)	35 (8.6)	0.770
Probably yes	190 (11.3)	147 (11.5)	43 (10.6)	
Yes	1357 (80.7)	1029 (80.7)	328 (80.8)	
Smoking				
Never smoked	1604 (95.4)	1219 (95.6)	385 (94.8)	0.608
Former smoker	33 (2.0)	24 (1.9)	9 (2.2)	
Current smoker	36 (2.1)	25 (2.0)	11 (2.7)	
(Missing)	8 (0.5)	7 (0.5)	1 (0.2)	
Alcohol use				
Non-users	1310 (77.9)	989 (77.6)	321 (79.1)	0.671
Light users	249 (14.8)	192 (15.1)	57 (14.0)	
Heavy users	95 (5.7)	75 (5.9)	20 (4.9)	
HIV test results at baseline				
No	1557 (92.6)	1205 (94.5)	352 (86.7)	1.000
HIV infection	79 (4.7)	61 (4.8)	18 (4.4)	
(Missing)	45 (2.7)	9 (0.7)	36 (8.9)	

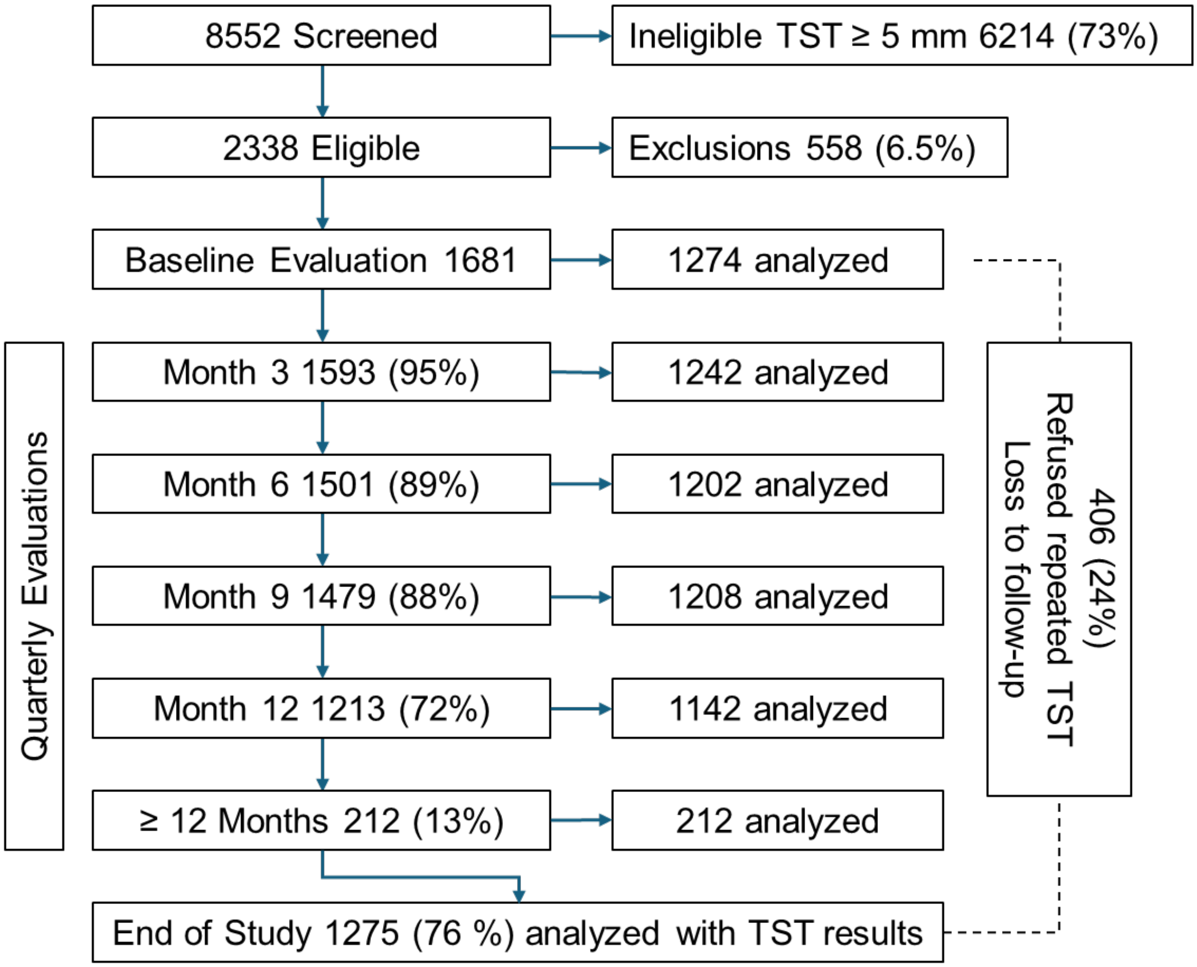

Figure S1. Study profile for recruiting and following participants in Kampala, Uganda, 2014 - 2017

(A)

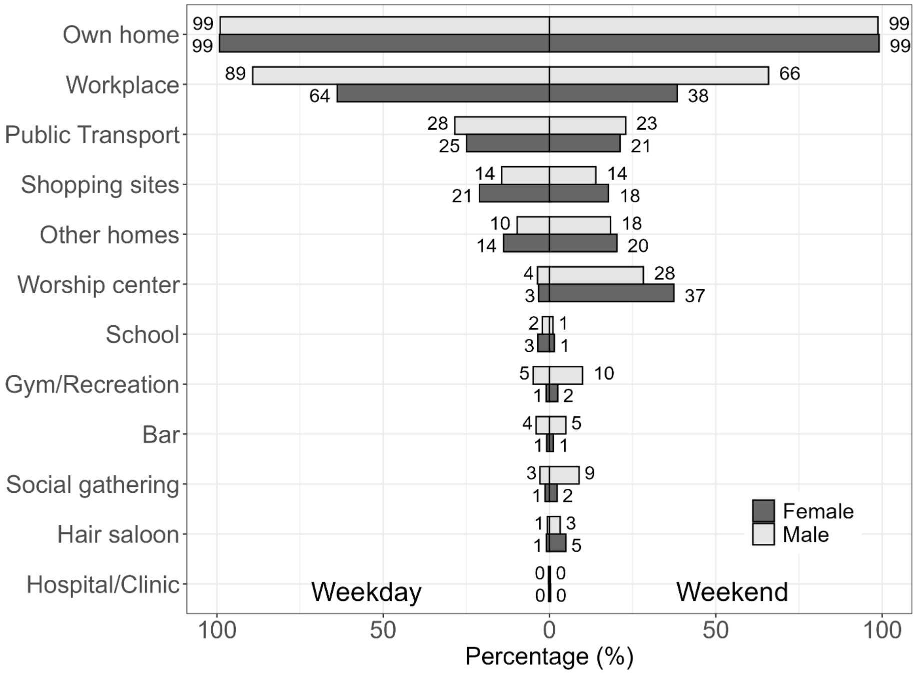

(B)

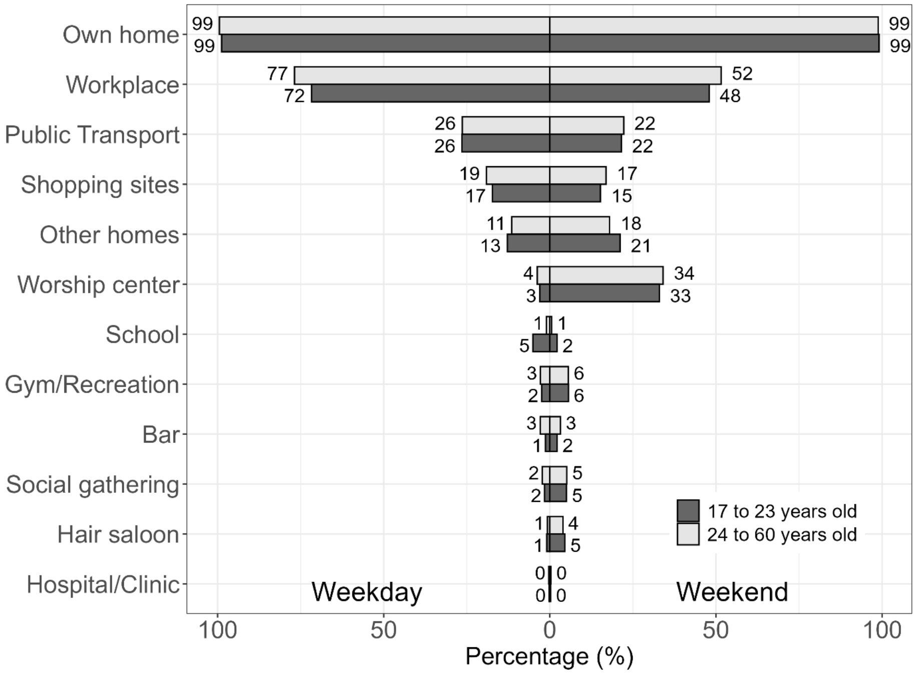

Figure S2. A. Percentage of evaluations when participants reported their attendance at a setting stratified by sex and weekday/weekend; B. Percentage of evaluations when participants reported their attendance at a setting stratified by age categories and weekday/weekend

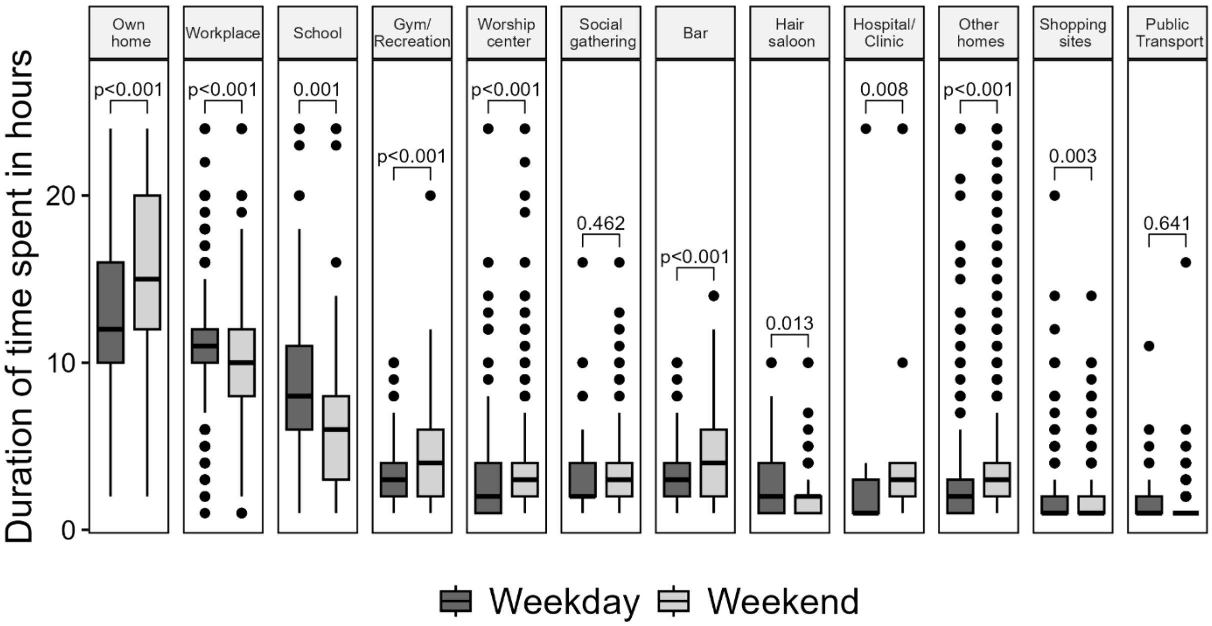

	Own home	Work place	School	Gym/ Recreation	Worship center	Social gathering	Bar	Hair salon	Hospital/ Clinic	Other homes	Shopping sites	Public transport
Weekday	1275	1212	111	139	167	103	86	46	16	528	659	866
Weekend	1275	1099	61	255	995	223	116	220	18	789	626	812

Figure S3. Difference in duration of time spent between weekdays and weekend when reported and the number of participants attended settings over entire period of observation

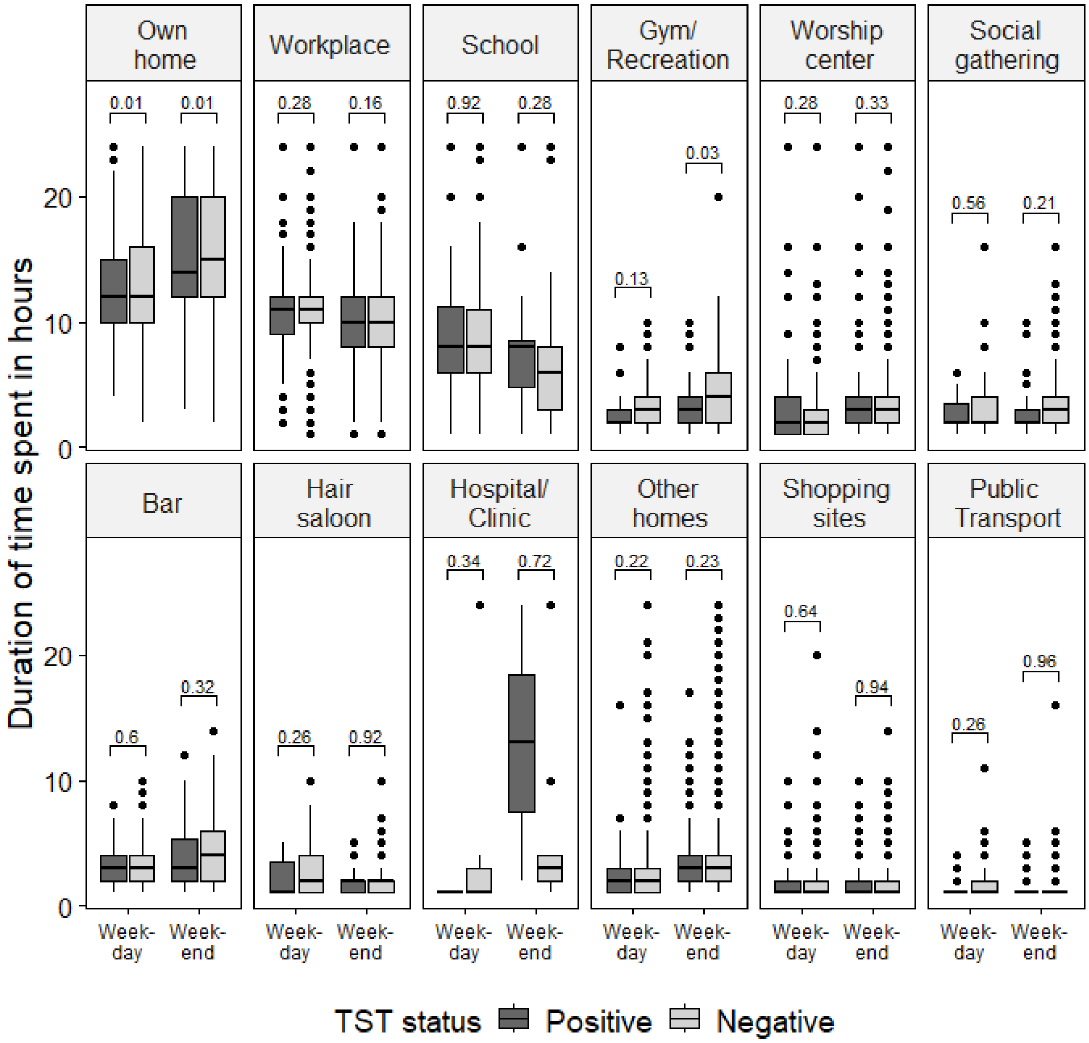

Figure S4. Difference in duration of time spent by TST status in weekdays and weekend when reported by participant

(A)

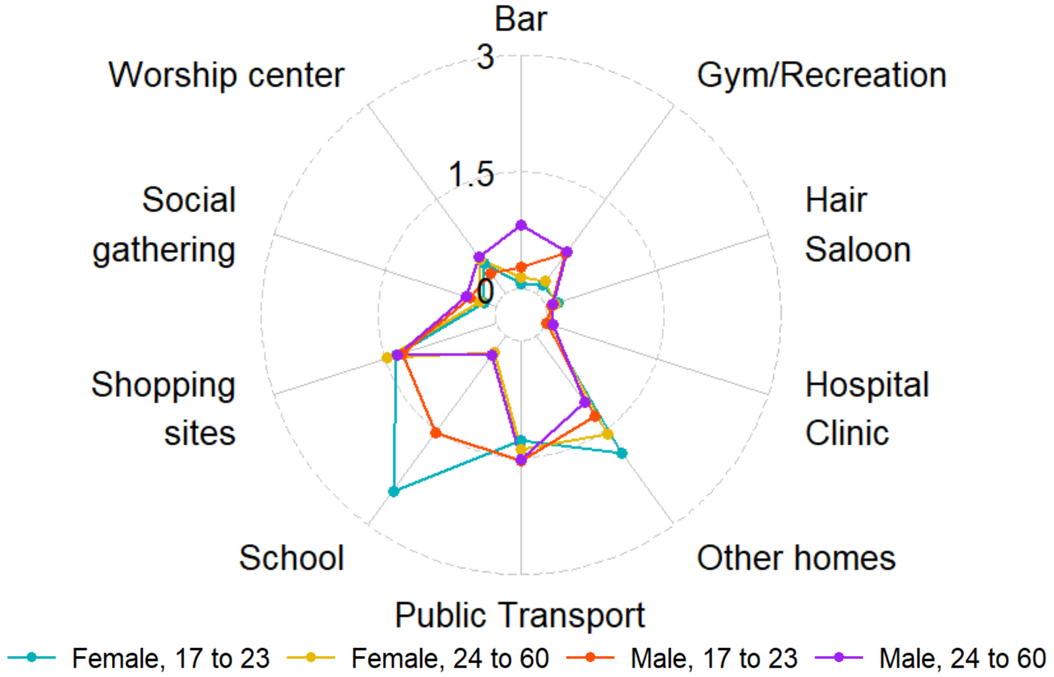

(B)

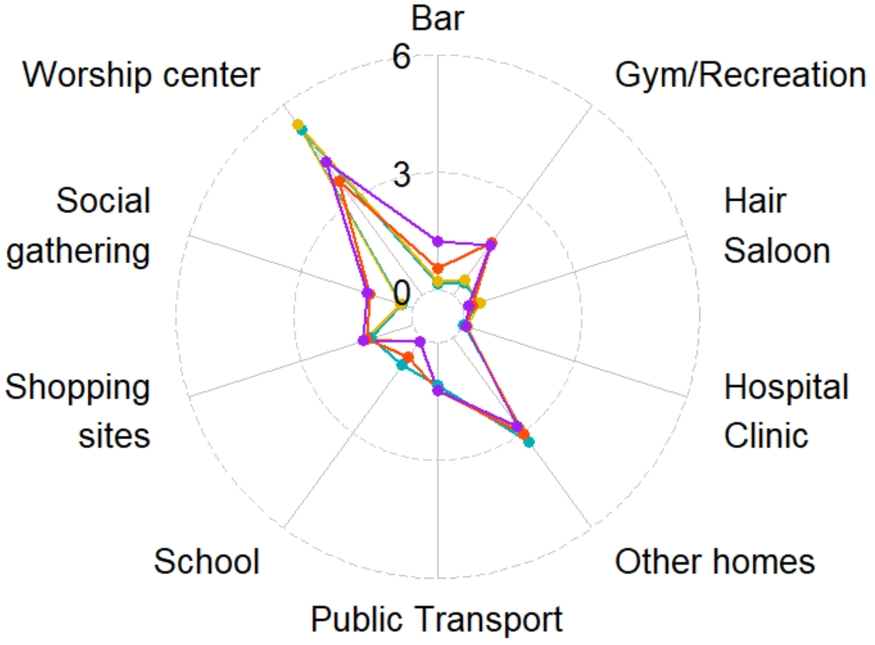

Figure S5. Radial graph showing the percent of the day spent at various settings on (A) weekdays and (B) weekend, stratified by age and sex

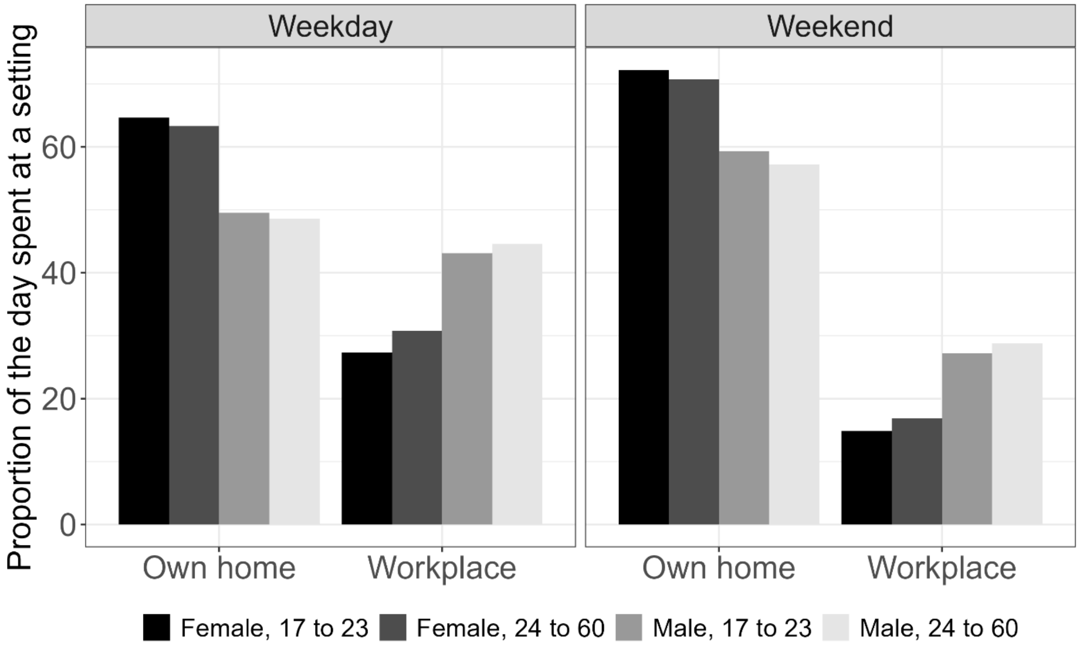

Figure S6. Percent of the day spent at own home and workplace stratified by age and sex

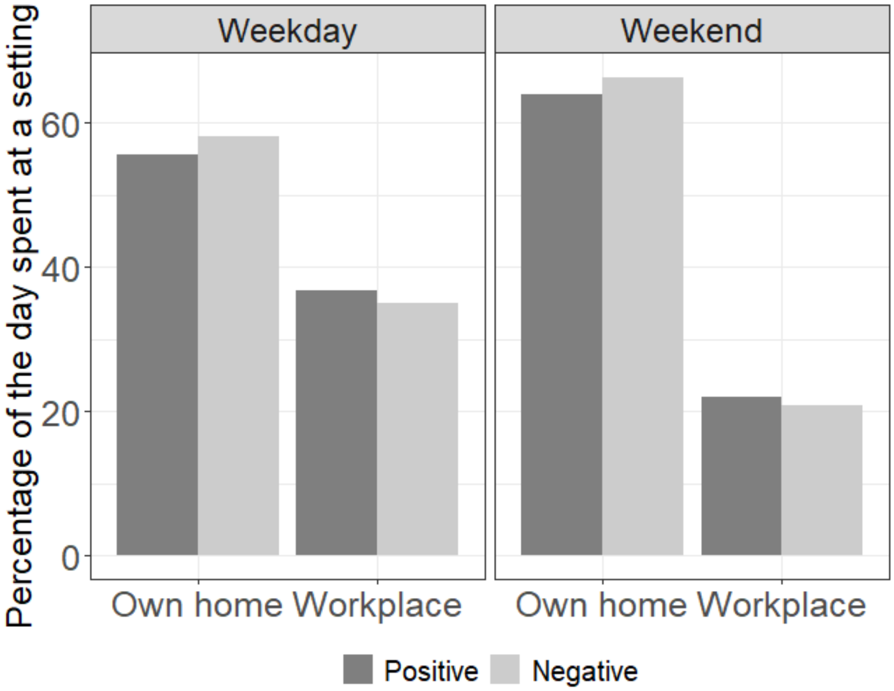

Figure S7. Percent of the day spent at own home and workplace stratified by TST status
